# Green fabrication of lanthanide-doped hydroxide-based phosphors: Y(OH)_3_:Eu^3+^ nanoparticles for white light generation

**DOI:** 10.3762/bjnano.10.119

**Published:** 2019-06-07

**Authors:** Tugrul Guner, Anilcan Kus, Mehmet Ozcan, Aziz Genc, Hasan Sahin, Mustafa M Demir

**Affiliations:** 1 Department of Materials Science and Engineering, Izmir Institute of Technology, Izmir, Turkey; 2 Department of Photonics, Izmir Institute of Technology, Izmir, Turkey; 3 Metallurgical and Materials Engineering Department, Faculty of Engineering, Bartin University, 74100 Bartin, Turkey

**Keywords:** green synthesis, phosphors, phosphor-converted white LED, white LED, white light generation

## Abstract

Phosphors can serve as color conversion layers to generate white light with varying optical features, including color rendering index (CRI), high correlated color temperature (CCT), and luminous efficacy. However, they are typically produced under harsh synthesis conditions such as high temperature, high pressure, and/or by employing a large amount of solvent. In this work, a facile, water-based, rapid method has been proposed to fabricate lanthanide-doped hydroxide-based phosphors. In this sense, sub-micrometer-sized Y(OH)_3_:Eu^3+^ particles (as red phosphor) were synthesized in water at ambient conditions in ≤60 min reaction time. The doping ratio was controlled from 2.5–20 mol %. Additionally, first principle calculations were performed on Y(OH)_3_:Eu^3+^ to understand the preferable doping scenario and its optoelectronic properties. As an application, these fabricated red phosphors were integrated into a PDMS/YAG:Ce^3+^ composite and used to generate white light. The resulting white light showed a remarkable improvement (≈24%) in terms of luminous efficiency, a slight reduction of CCT (from 3900 to 3600 K), and an unchanged CRI (≈60) as the amount of Y(OH)_3_:Eu^3+^ was increased.

## Introduction

Interior lighting is an integral part of civilization, and its development has evolved toward the design of more energy efficient technologies [1–5]. Today, light-emitting diode (LED)-based white light generation plays a role in both academic and industrial applications. To obtain white light through LED-based configurations, either all three primary colors (red–green–blue (RGB)) can be integrated individually via LED chips or various luminescent materials can be used as color conversion layers over blue or UV LED chips [6]. In this sense, phosphors are commonly used luminescent materials applied as color conversion layers due to their optical features and high stability [7–13]. Among those, cerium-doped yttrium aluminum garnet (YAG:Ce^3+^) is a well-known yellow phosphor that is employed in an LED package together with a blue LED to create a white light LED (WLED) [14–19]. In such a case, the blue light emitted from the blue LED impinges on the phosphor-containing color conversion layer. Some portion of the blue light is absorbed by the phosphor and yellow emission is produced. Meanwhile, the remaining portion of the blue light escapes from this layer. The combination of the escaped blue light and the produced yellow emission from the phosphor from the conversion layer results in the generation of white light.

WLED systems composed of a blue LED and YAG:Ce^3+^ is a facile and inexpensive way of obtaining white light. However, the white light by this system displays some inadequate optical properties, such as high correlated color temperature (CCT) and low color rendering index (CRI) due to a deficiency in red light [20–21]. To overcome this problem, one can integrate additional phosphors, such as red or red and green phosphors over the blue LED or one can employ the primary RGB colors over a UV or near-UV LED chip or one can use alternative luminescent materials, such as quantum dots [22], perovskites [23], organic dyes [24], etc. In the case of phosphors, combining a red phosphor with YAG:Ce^3+^ over a blue LED is the simplest way of increasing the CRI while reducing the CCT.

Phosphors mostly consist of thermally and chemically stable inorganic hosts such as YAG combined with rare-earth dopants (Ce^3+^, Eu^3+^, Dy^3+^, etc.) [9–11,13]. Visible range emission from these phosphors, such as yellow, green or red, are in general the result of radiative energy transfer between partially filled 4f orbitals of dopant states together with the effective shielding of 5s and 5p orbitals [25]. Moreover, the photoluminescence (PL) intensity depends on the transition between 4f → 4f states; the transition from ^5^D_0_ to ^7^F_1_, ^5^D_0_ to ^7^F_3_ and ^5^D_0_ to ^7^F_4_ results in low intensity, while the transition from ^5^D_0_ to ^7^F_2_ leads to high PL intensity. Therefore, by manipulating the energy levels of the transition states through adjusting the dopant ion, different emission and PL intensities can be obtained [11,26–28]. These phosphors have been employed in various applications, including optoelectronics, field emissive displays and HDTVs, and advanced ceramics [29–30].

To date, numerous methods for obtaining phosphors to be used either over blue LED or UV LED have been reported. However, these materials require harsh synthesis conditions such as high temperature and high pressure, and water-free solvents, which can restrict their commercialization. In this context, several methods have been widely used such as sol–gel, hydrothermal, combustion, emulsion, and precipitation methods [31–34]. Among those, the sol–gel and co-precipitation methods are, in general, slow and usually involve additional steps. On the other hand, there is a huge waste of organic solvents during the emulsion process, which makes this method inefficient in terms of cost and toxicity [35]. Therefore, facile synthesis methods involving water-based reactions at ambient conditions are needed for phosphor fabrication. In this study, a promising strategy has been introduced in order to meet these requirements especially in the case of lanthanide-doped hydroxide-based phosphor fabrication, which is facile, water-based, and rapid. More specifically, acetate-based reagents of both the host and dopant are dissolved in LiOH/water solution together under room temperature. The presence of Li ions during the reaction process may distort the crystal structure and lead to an increase in the formation of substitutional defects, which may facilitate the incorporation of dopant ions into the system [36–39]. As an example, luminescent red Y(OH)_3_:Eu^3+^ phosphors were fabricated via employing this method. The doping process and complete crystallization were achieved in 60 min. State-of-the-art first-principle calculations were performed on Y(OH)_3_:Eu^3+^ to investigate its crystallographic structure and resulting electronic and optical properties. In summary, a novel water-based, rapid, and simple method was developed in this study. As an application, the red emitting phosphor has been fabricated at ambient conditions in a short time. This method can be a promising strategy for fabricating phosphors to be employed as down-converting materials for pc-converted WLED systems [33,40–43].

## Experimental

### Materials and methods

Yttrium(III) acetate hydrate (Y(Ac)_3_·xH_2_O; >99%), europium(III) acetate hydrate (Eu(Ac)_3_·H_2_O; >99%), and lithium hydroxide (LiOH; 98%) were purchased from Sigma-Aldrich (St. Louis, MO, USA) and used as-received without any further purification. Cerium-doped yttrium aluminum garnet (YAG:Ce^3+^, HB-4155H, Zhuhai HanboTrading Co., Ltd., Guangdong, China) was used as a yellow phosphor and polydimethylsiloxane (PDMS) (SYLGARD 184 Kit, Dow Corning, Midland, MI, USA) was used as the polymer matrix. The crystallographic properties of the crystals were characterized by X-ray diffraction (XRD; X’Pert Pro, Philips, Eindhoven, The Netherlands), while their morphology was characterized by scanning electron microscopy (SEM; Quanta 250, FEI, Hillsboro, OR, USA). High-resolution transmission electron microscopy (HRTEM) micrographs were obtained using an FEI Tecnai F20 field emission gun microscope with a 0.19 nm point-to-point resolution at 200 keV equipped with an embedded Quantum Gatan Image Filter (Quantum GIF) for electron energy loss spectroscopy (EELS) analysis. The images were analyzed via Gatan Digital Micrograph software. Optical characterization was carried out using an Ocean Optics spectrometer (Ocean Optics, USB2000+, Duiven, The Netherlands).

### Synthesis of Eu-doped yttrium hydroxide crystals

An amount of yttrium(III) acetate hydrate (5.63 × 10^−4^ mol) and a varying amount of europium(III) acetate hydrate were dissolved in 10 mL of deionized water. Depending on the dopant ratio, the amount of europium(III) acetate hydrate was added (for instance, to achieve 7.5% Eu^3+^ dopant ratio, 4.55 × 10^−5^ mol of europium(III) acetate hydrate was employed). Subsequently, the mixture was stirred in a glass container until it appeared transparent. An amount of LiOH (0.04 mol) was added into the transparent solution, respectively. Selecting LiOH as the ion source is critical here since the other possible ions such as Na^+^ and K^+^ are not as reactive as Li^+^ ions. Such a high reactivity of Li^+^ ions in the solution is expected to distort the crystal structure relatively more compared to other possible ions and can therefore lead to an increase in the formation of defects to make the doping process more favorable (as previously mentioned at the end of the Introduction section). The solutions were mixed and sonicated for 5 min. After the sonication process, the reaction was maintained for various reaction times (5, 15, 45, 60 min) at room temperature. The reaction mixture was centrifuged twice with water and once with ethanol (5 min, 6000 rpm). After centrifugation, the isolated products were dried in an oven at 100 °C for 1 h.

### Preparation of PDMS/Eu-doped yttrium hydroxide composites

1 g of PDMS (composed of a silicon elastomer and curing agent with 10:1 ratio) was prepared in a glass vial. Then, a fixed amount (70.0 mg) of yellow YAG:Ce^3+^ phosphor was added into the glass vial. In order to investigate the effect of the red phosphor amount on the resulting white light properties, a desired amount of the red Y(OH)_3_:Eu^3+^ sub-micrometer phosphor in mass was introduced into the PDMS/YAG:Ce^3+^. The resulting mixture was stirred until all phosphors mixed homogenously. Then, this mixture was dropped into a mold having a fixed thickness of 0.2 cm and a diameter of 2.0 cm and the mold was put into a vacuum oven for 30 min for solvent evaporation. Finally, this mold was cured and cross-linked at 100 °C for 30 min and then the free-standing composite film was removed from the mold.

### Computational details

To investigate the effect of europium dopants on the structural and electronic properties of Y(OH)_3_ crystals, density functional theory-based calculations were also performed using the projector augmented wave (PAW) potentials as implemented in the Vienna ab initio simulation package (VASP) [44–47]. For the exchange-correlation part of the functional, the generalized gradient approximation (GGA) in the Perdew–Burke–Ernzerhof (PBE) form was employed [48]. In order to obtain the charge transfer between the atoms, the Bader technique was used [49]. The kinetic energy cut-off for plane-wave basis set was taken as 400 eV for all the calculations. For all ionic relaxations, the total energy difference between the sequential steps in the calculations was taken to be 10^−5^ eV as the convergence criterion. On each unit cell, the total forces were reduced to a value less than 10^−4^ eV/Å. Г-centered k-point meshes of 2 × 2 × 2 were used for a 128-atom supercell of bulk Y(OH)_3_.

## Results and Discussion

### Structural characterization of Y(OH)_3_:Eu^3+^ particles

**Figure 1:**
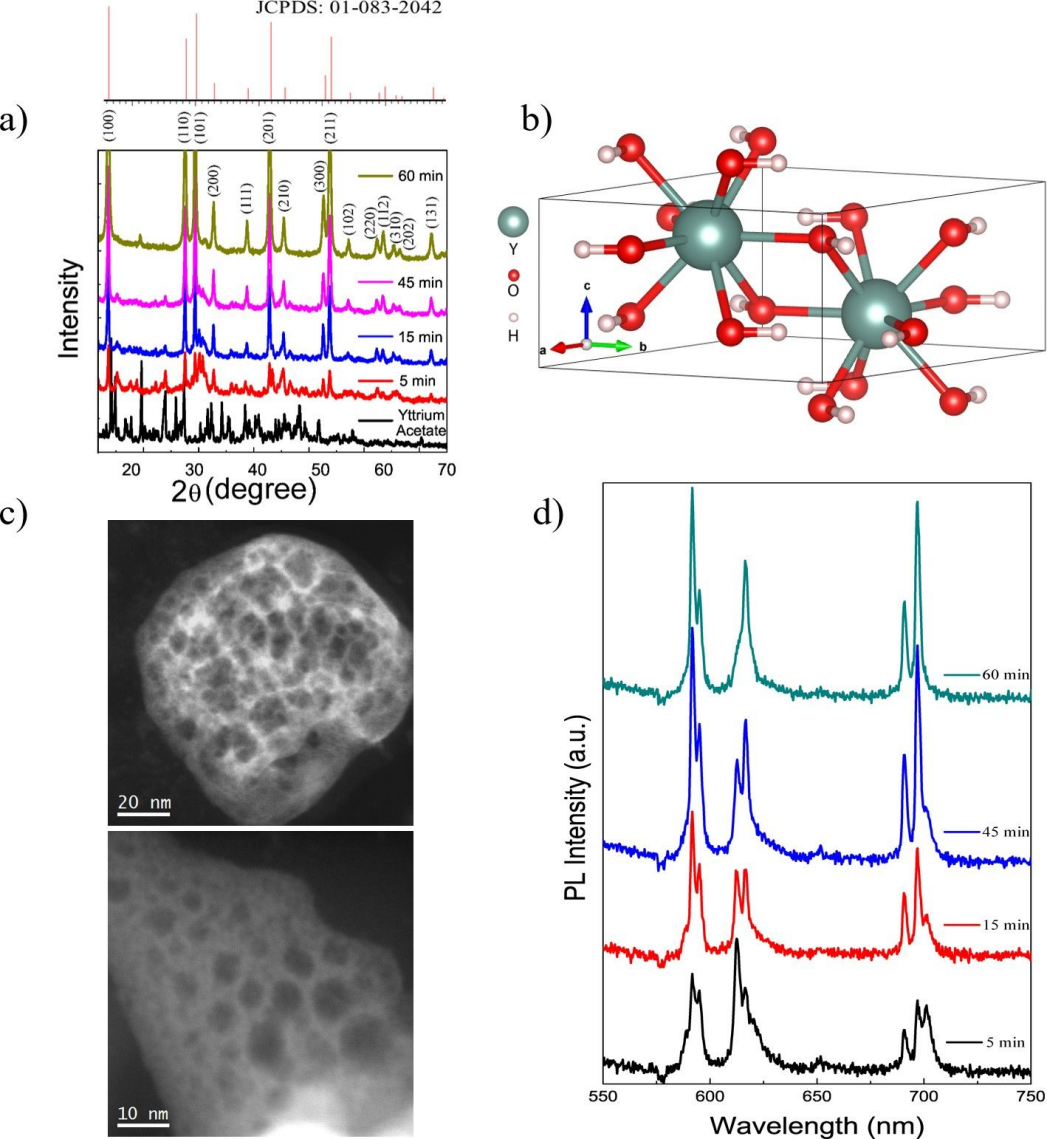
(a) XRD patterns of Y(OH)_3_:7.5% Eu^3+^ phosphors prepared at various reaction times: 5, 15, 45, and 60 min, (b) schematic presentation of the unit cell of Y(OH)_3_ lattice, (c) general HAADF-STEM micrographs of the Y(OH)_3_:(7.5% Eu^3+^) particles prepared at 5 min showing the presence agglomerated nanocrystals, and (d) the photoluminescence (PL) spectrum of Y(OH)_3_:7.5% Eu^3+^ phosphors.

Figure 1a presents the X-ray diffraction pattern of representative Y(OH)_3_:Eu^3+^ particles having 7.5% dopant ratio (Y(OH)_3_:7.5% Eu^3+^) prepared at various reaction times. The pattern of the starting material (i.e., unreacted yttrium acetate) is given for the comparison. The reflections can be indexed to hexagonal phase (space group of P63/m) Y(OH)_3_ with lattice constants of *a* = 6.261 Å, *b* = 6.261 Å, and *c* = 3.544 Å corresponding to JCPDS: 01-083-2042. The evolution of the crystal is clearly seen in the stack plot of the patterns. When the yttrium acetate is treated with alkaline water for 5 min its reflections begin to disappear. The extension of the synthesis time to 15 min reduces the intensity of the precursor reflections, at the end of 45 min, and characteristic signals of the Y(OH)_3_ labeled with their corresponding planes become more evident. After 60 min of reaction time, the reflection signals of the resulting product were found to be perfectly matched with the crystallographic data of JCPDS: 01-083-2042. The primitive unit cell of the resulting Y(OH)_3_ host crystal is presented in Figure 1b. This crystal structure indicates two yttrium atoms located at one face of the hexagonal phase and shared oxygen saturated with hydrogens. To investigate the crystal formation of the samples fabricated at 5 min, several low-magnification high-angle annular dark field (HAADF) STEM micrographs were taken and are presented in Figure 1c. These micrographs reveal that the sample consists of agglomerated nanoparticles. The clusters formed by the sub-10 nm nanoparticles have sizes between 50 nm to a few micrometers. It is possible to visualize the individual nanoparticles in the lower left HAADF STEM micrograph where the building blocks of these agglomerates seem to have sizes smaller than 5 nm. The photoluminescence spectrum of the Y(OH)_3_:7.5% Eu^3+^ phosphors that were synthesized at varying times was registered at 365 nm excitation wavelength (Figure 1d). At the first 5 min, sharp emission signals appear at 592, 595, 613, 616, 690, 697 and 700 nm, indicating specific D–D and D–F transitions of Eu^3+^ states may be due to crystal splitting by the Y(OH)_3_ host. As the synthesis time is extended, the intensity of the signals at 592 and 697 nm shows a significant increase while the ones at 613 and 700 nm diminish upon crystallization.

**Figure 2:**
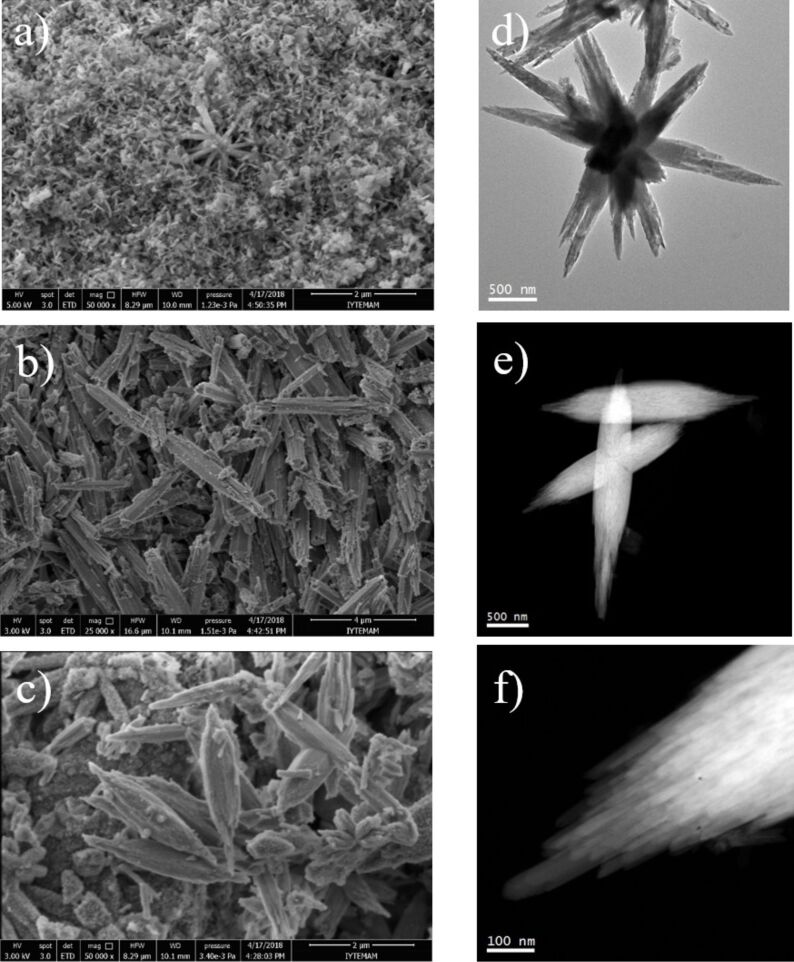
SEM images of the Y(OH)_3_:7.5% Eu^3+^ phosphors obtained at different synthesis times: (a) 5 min, (b) 15 min, and (c) 60 min. (d–f) Overview TEM and HAADF-STEM micrographs of the particles prepared at 60 min. The sample is composed of micrometer-sized multipods, which seem to be a controlled ensemble of ≈10 nm thick nanowires.

The SEM images demonstrate the morphology of the Y(OH)_3_:7.5% Eu^3+^ crystals fabricated at varying times in Figure 2a–c, from 5 to 60 min, respectively. The change in morphology of the crystals is evident. The phosphors obtained at 5 min of reaction time show a needle-like shape with nanometer-scale size distribution (Figure 2a). After 15 min of reaction, the crystals grow larger and started to show a rod-like structure with sub-micrometer sizes (Figure 2b). As the reaction time is extended to 60 min, the crystals transform into a rice-like structure (Figure 2c). For more detailed information about the morphology of Y(OH)_3_:7.5% Eu^3+^ crystals, general TEM and HAADF-STEM micrographs of the sample prepared at 60 min are presented in Figure 2d–f. A multipod-like structure (Figure 2d) together with the rice-like structures (Figure 2e,f) were obtained. Higher magnification images, as presented in Figure 2e, indicate that the multipod-like structures are the result of the reunion of rice-like structures. On the other hand, the higher magnification images on the rice-like shapes demonstrate that these structures have fringed edges (Figure 2f), which may be the ensemble of ≈10 nm thick individual nanowires.

**Figure 3:**
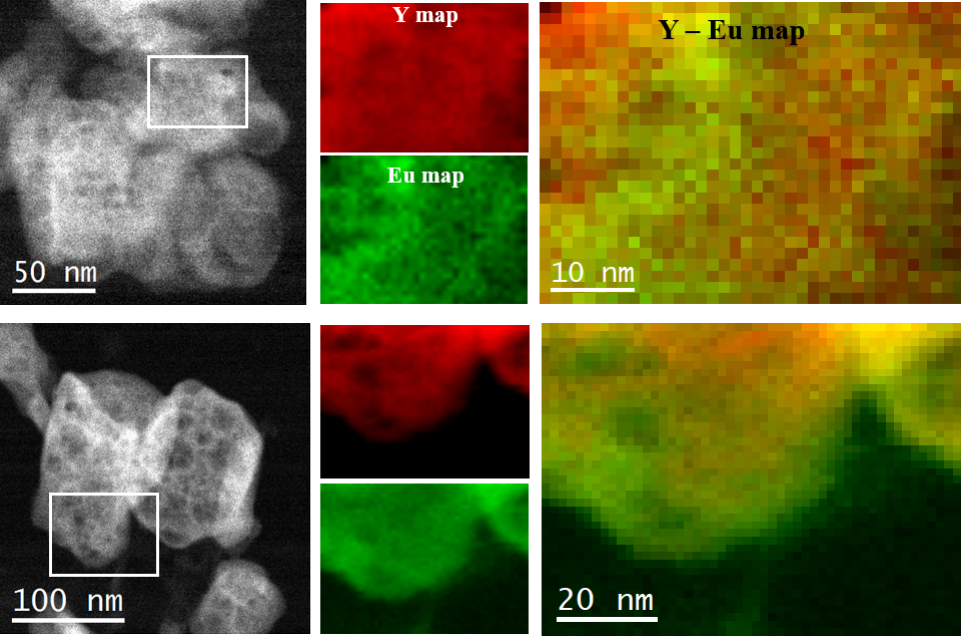
Annular dark field (ADF) STEM micrograph of an agglomerate of nanoparticles synthesized at 5 min with 7.5% doping ratio. STEM-EELS elemental composition maps of the area indicated with a white rectangle: Y (in red) and Eu (in green) maps along with their composite image.

The level of doping for Y(OH)_3_:7.5% Eu^3+^ phosphors produced at 5 min was captured through an annular dark field (ADF) STEM micrograph and the STEM-EELS analysis of the indicated area is presented in Figure 3. Elemental composition maps of Y (red) and Eu (green) along with their composites are shown. (Experimental note: The above presented maps are obtained from the Eu M_5,4_ edges located at 1131 eV and 1161 eV and Y L3,2 edges located at 2080 eV and 2155 eV by using a 1 eV/channel.) The elements Y and Eu exhibit an even distribution throughout the nanoparticle volume with some presence of slightly Eu-rich or Y-rich regions.

**Figure 4:**
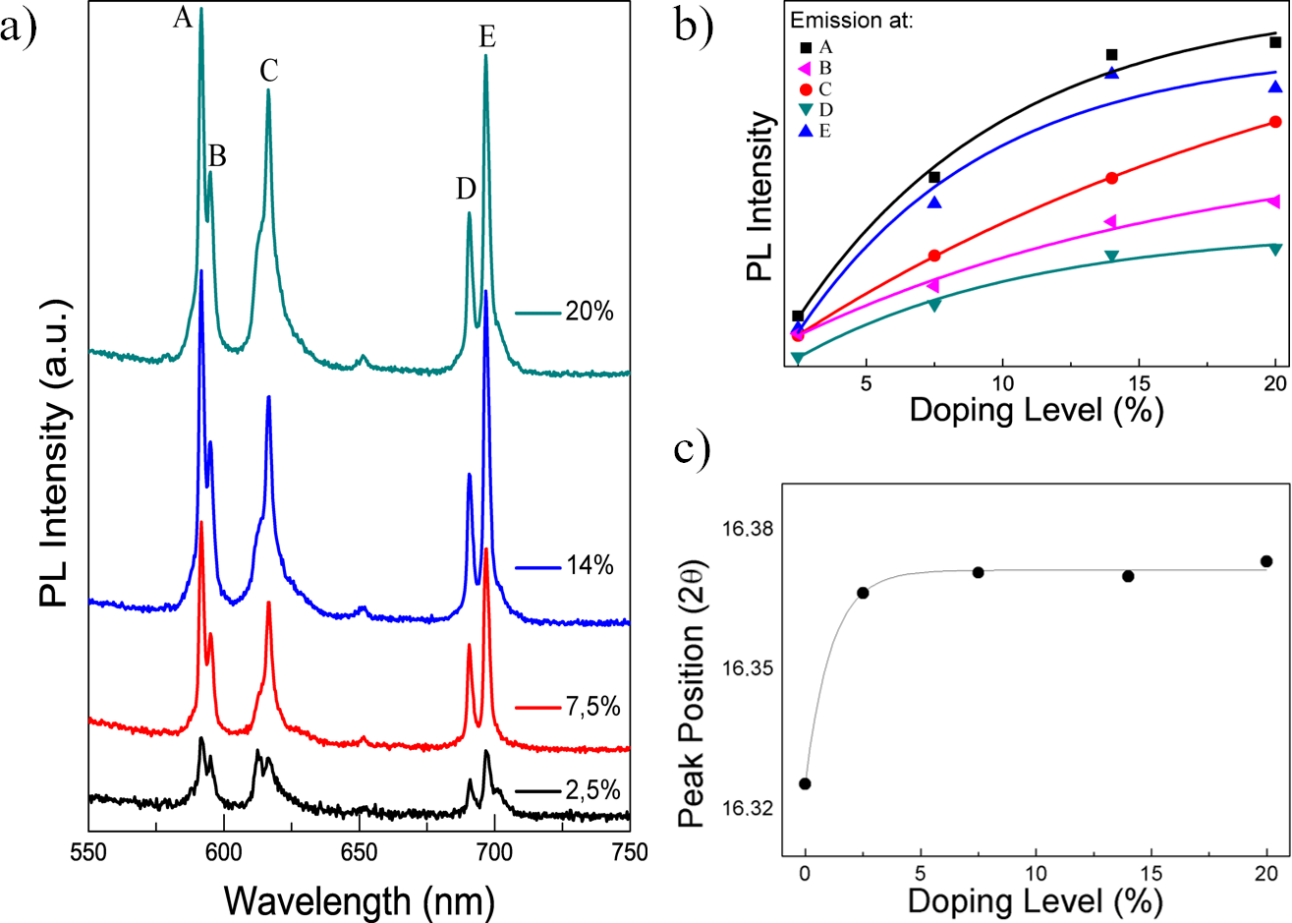
(a) Photoluminescence (PL) spectrum of the Y(OH)_3_:Eu^3+^ phosphors synthesized for 60 min with different doping ratios. (b) Variation of their corresponding emission peak intensities, labeled with A, B, C, D, and E, with respect to doping ratio. (c) The shift of the 2θ reflection position of 16° with respect to the doping ratio of the particles.

The effect of the doping ratio on the photoluminescence properties was investigated. Figure 4a presents the PL spectra of the Y(OH)_3_:Eu^3+^ red phosphors fabricated at 60 min having various dopant ratios (λ_Exc_ = 365 nm). Characteristic signals of the corresponding transition states of the Eu^3+^ ion are labeled with A, B, C, D and E to be able to track their changes with respect to adjusted dopant ratios. Initially, at the doping ratio of 2.5%, these signals are comparable with each other in terms of their PL intensity. However, as the doping ratio increases, all emission signals, especially the signals corresponding to A, C, and E, show a significant increase. The increase of the emission signals of A, C, and E show a trend to grow faster than the remaining B and D points. The change of these corresponding emission intensities as a function of doping ratio is presented in Figure 4b. In any case, these values are saturating with respect to doping ratio, which is expected since the possibility of incorporation of Eu ions into Y(OH)_3_ is limited. This limit can be inferred from the Figure 4b as 25–30%. The effect of the doping ratio on the XRD reflection signals was explored. Since atomic size of Y and Eu is different, the highest reflection at 16° (2θ), corresponding to the (100) plane, was examined to determine if any shift is present for the samples of all doping ratios. The crystals that were prepared in 60 min were considered, and their peak positions as a function of the doping ratio are demonstrated in Figure 4c. The corresponding reflections at 16° are fitted with a Gaussian distribution, and then their exact location was registered. As the doping ratio increases, a slight shift of the reflections is observed. This shift may be the result of enlarging the lattice of the host crystal due to incorporation of Eu^3+^ ions (since it has a larger ionic radius (0.109 nm) compared to Y^3+^ ions (0.104 nm)) [50–51].

### Proposed growth mechanism of Y(OH)_3_:Eu^3+^ crystals

**Scheme 1:**
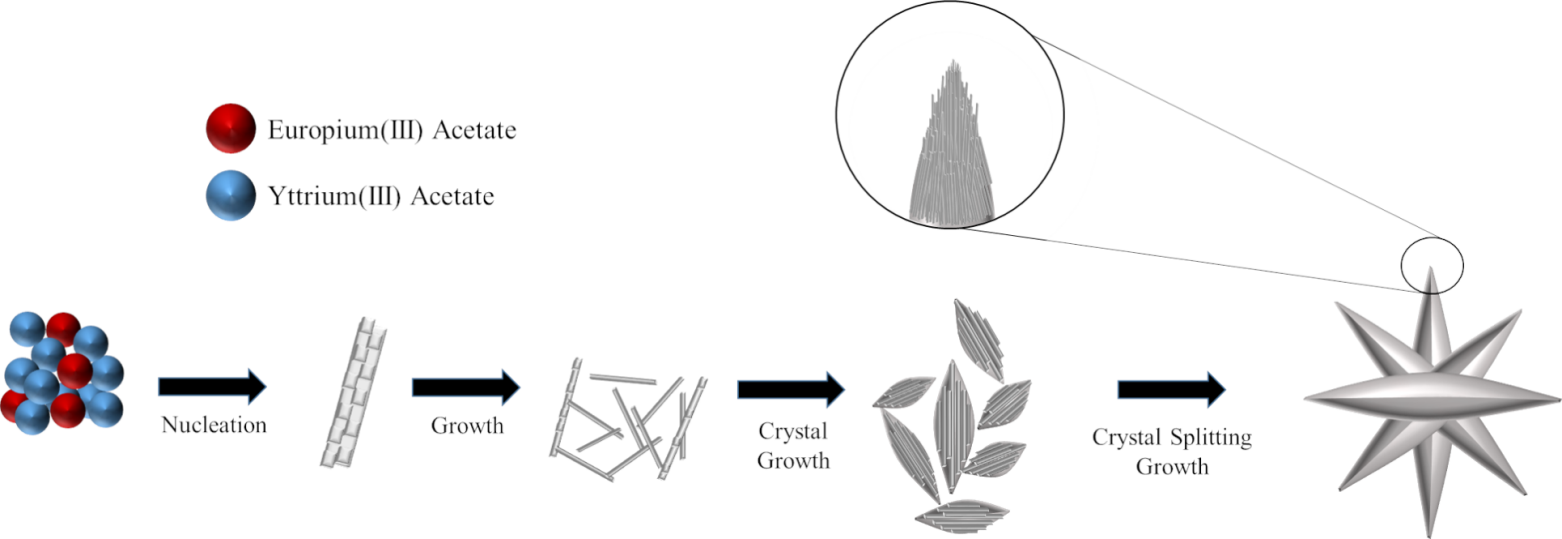
Schematic of the crystal growth mechanism for the development of the multipod-like structure of the Y(OH)_3_:Eu^3+^ phosphors.

The growth mechanism of the resulting Y(OH)_3_:Eu^3+^ crystals was investigated by considering both the SEM and TEM images (Figure 1c and Figure 2). The growth mechanism that governs almost the entire nucleation and crystal growth process here was reported already by Hussain et al. [52] in detail, where the authors employed hexamethylenetetramine (HMTA) during the fabrication of La(OH)_3_:Eu^3+^ crystals. HMTA degrades into ammonia that acts as hydroxide source. In this study, HMTA was replaced with LiOH, and Y(OH)_3_:Eu^3+^ crystals were obtained through the interaction of OH^−^ ions released from LiOH with Y^3+^ ions from the yttrium source in water at room temperature. Even at 5 min, Y(OH)_3_:7.5% Eu^3+^ crystals were formed as in a needle-like shape. Such a fast crystal formation may indicate fast nucleation, and here (which is similar to the idea proposed by Hussain et al. [52] where the authors argued that the excessive NH_4_^+^ ions may accelerate the reaction between La^3+^ and OH^−^), excessive Li^+^ ions in the reaction media may be responsible for this rapid growth. Extending the reaction time to 15 min, the crystals grow into larger structures and rod-like structures appear in the form of nanorod bundles, probably due to crystal splitting. For 60 min of reaction time, rod-like structures transform into rice-like shapes that are in contact mainly with each other as a part of a self-assembly process occurring as a result of a saturated splitting process [52]. Moreover, multipod-like (or flower-like) morphology was also observed for some particular crystals, which are probably the result of this self-assembly process where there is more time to act on these particular crystals. The crystal growth mechanism is summarized and illustrated in Scheme 1. Compared to HMTA, which decomposes into ammonia and releases OH^−^ ions slowly, LiOH is able to give OH^−^ ions through complete dissociation directly to the reaction medium. Since the rate of hydroxide release is a strong parameter to control the size, size distribution and defect content of the resulting crystal, it is expected to obtain a variation for these values in the case of comparing the effect of HMTA and LiOH on the crystal growth. Moreover, such a difference between HMTA and LiOH in terms of the rate of OH^−^ release may allow the formation of these crystals even at room temperature (as reported in this study), while the authors that used HMTA kept the reaction mixture at 75 °C. On the other hand, crystal growth splitting, as observed for various material systems such as SrTiO_3_ [53], Zn_2_GeO_4_ [54], La(OH)_3_:Eu^3+^ and La_2_O_3_:Eu^3+^ [52] in the literature, is associated with fast crystal growth. A possible cause could be the oversaturation of reactant species. When the concentration of reactive species appears to be higher than the threshold (which may vary for each material depending on its chemistry), fast crystal growth takes place. This fast growth may force a high density of crystal defects, i.e., the atoms do not have enough time for placement in the crystal array and the metal atom misplacement may occur during the fast growth. These defects gradually develop nuclei sites developing branches, leading eventually to splitting.

### First principle calculations of Y(OH)_3_:Eu^3+^

**Figure 5:**
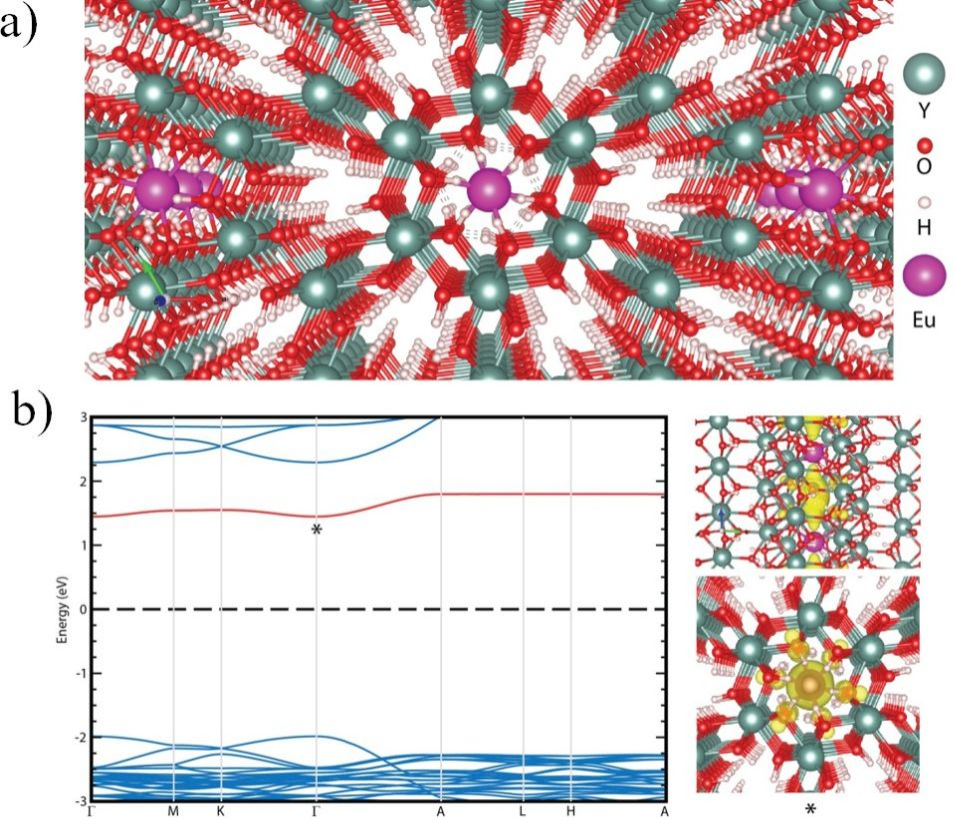
(a) Perspective view of the atomic structure of Eu-doped Y(OH)_3_. (b) (left) The electronic band dispersion of Eu-doped Y(OH)_3_ (the Fermi level is set to zero) and (right) top and side view of the charge density corresponding to the midgap electronic state.

Our calculations reveal that the ground state structure of Y(OH)_3_ has a hexagonal crystal symmetry with space group P63/m. Figure 5a demonstrates the 14-atom primitive unit cell of Y(OH)_3_ which consists of 2 yttrium, 6 oxygen and 6 hydrogen atoms. The optimized lattice parameters of bulk Y(OH)_3_ are found to be *a* = 6.10 Å, *b* = 6.10 Å and *c* = 3.51 Å. In this structure each Y atom bonds with nine O atoms with a bond length of 2.39 Å. According to the Pauling scale electronegativity of Y, H and O are 1.22, 2.20 and 3.44, respectively. Bader charge analysis shows that the Y(OH)_3_ crystal structure is formed by 0.73 (0.60) *e* charge transfer from the Y (H) to the O atom.

Possible scenarios for Eu doping in a Y(OH)_3_ crystal is also investigated by state-of-the-art first principles calculations. Total energy minimization calculations suggest that while substitutional doping of Eu atoms by Y, O or H is energetically unfavorable, interstitial doping at the holey site surrounded by the H atoms is a preferable adsorption site (Figure 5a). It was observed that the interstitial doping of Eu atoms slightly enlarges (0.5%) the bonds between neighboring atoms belonging to the Y(OH)_3_ crystal. Regarding the stability or robustness of Eu dopants in the host material, molecular dynamics calculations show that Eu atoms, covalently attached to the host lattice with a binding energy of 2.34 eV, maintain their atomic position in the Y(OH)_3_ crystal for more than 5 ps at room temperature.

Theoretical calculations show that the electronic structure of the Y(OH)_3_ host is also significantly modified by the Eu dopant. While the GGA-PBE approximated electronic band dispersion of the host material has a bandgap of 3.83 eV, some midgap states emerge after the interstitial doping of Eu. The energy bandgap of the host at the vicinity of doped region increases to 4.28 eV. The band and orbital decomposed charge density presented in Figure 5b shows that the midgap state is formed by strongly hybridized Eu and surrounding the O atoms. It appears that the strongly bonded Eu atoms not only lead to deformation in the lattice structure but also to emergence of defect-like states resulting in additional peaks in the PL spectrum.

### White light generation by integrating red Y(OH)_3_:Eu^3+^ phosphors into a YAG-based color conversion layer

**Figure 6:**
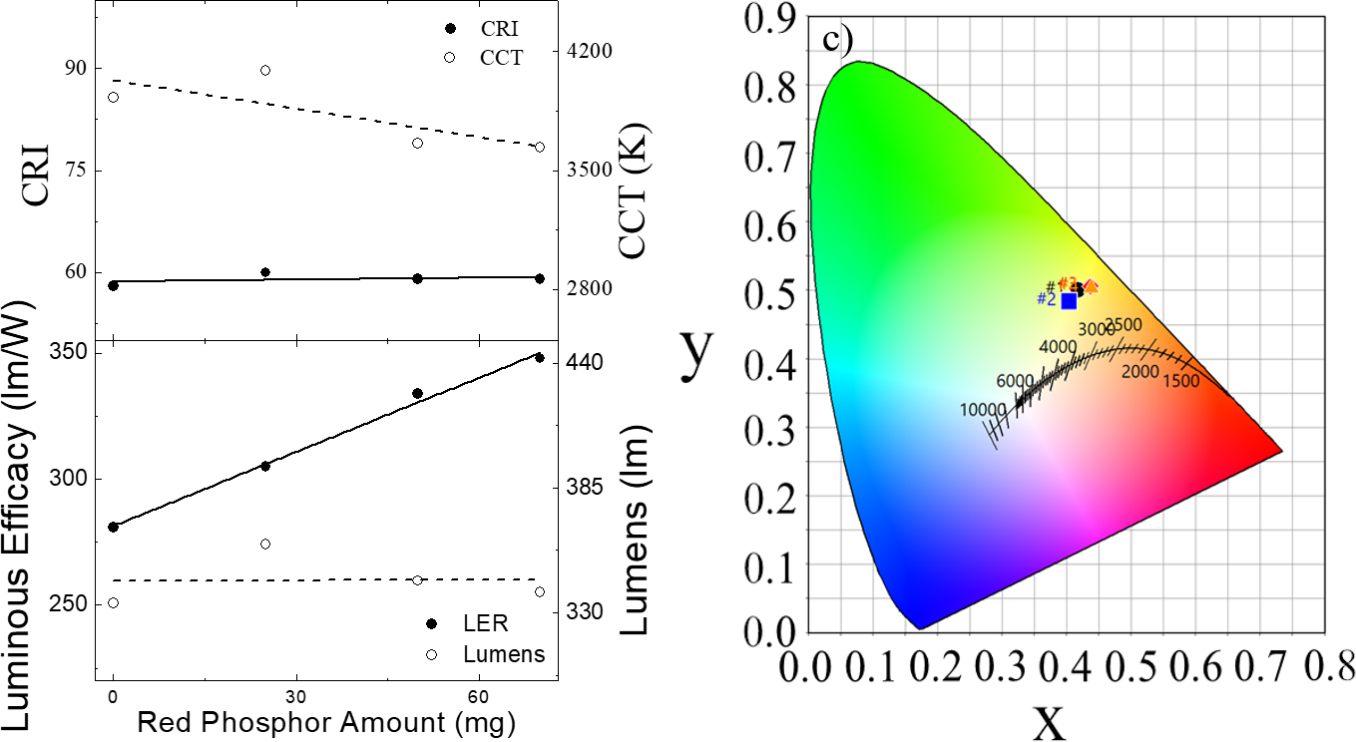
White LED application using a varying amount of red Y(OH)_3_:Eu^3+^ phosphors (fabricated at 60 min with a 20% doping ratio) combined with YAG:Ce^3+^ phosphor and their corresponding (a) CRI, CCT, (b) luminous efficacy (LER), lumens, and (c) CIE color coordinates.

Red-emitting Y(OH)_3_:Eu^3+^ sub-micrometer phosphors were integrated into a YAG-based white LED configuration, and the resulting optical features were investigated. Among the varying doping ratios, Y(OH)_3_:20% Eu^3+^ fabricated at 60 min was selected as a model material since the results from Figure 4a indicated that this sample had the highest emission intensity. The optical properties (CRI, CCT, luminous efficiency (LER), and lumen) of the PDMS composites are presented in Figure 6. Figure 6a shows the CRI and CCT of the PDMS composites as a function of the amount of red phosphor. While the CRI remains almost unchanged at around 60, the CCT decreases from 3900 to 3600 K. The main reason for the unfavorable CRI for this system may be the lack of blue color. On the other hand, a significant improvement from 281 to 348 lm/W (nearly a 24% increase) is observed for the luminous efficiency when the red phosphor is employed (Figure 6b). Meanwhile, the measurement of lumens remains almost the same, showing no significant change as the red phosphor amount increases. According to the color coordinates as presented in Figure 6c, all PDMS composites seem to be accumulated in the yellow region of the spectrum. They are far from the ideal white light and require more blue color to shift the resulting color spectrum towards white. Therefore, integration of Y(OH)_3_:20% Eu^3+^ into the PDMS/YAG:Ce^3+^ composite is just part of the process towards obtaining high quality white light generation. It is possible to obtain the primary colors, green, and blue by using the method proposed in this study by adjusting the lanthanide chemistry. In such a case, by combining all the primary colors, one can successfully generate high-quality white light with a high CRI and low CCT. Moreover, the efficiency of these systems can be improved even more by optimizing the long-term stability of PDMS under UV irradiation since it can undergo degradation due to the breakage of bonds in organic molecules, resulting in the loss of transparency and coloration (i.e., yellowing). For instance, it was observed that residues of PDMS can show a yellowish appearance and also embrittlement and cracking upon UV exposure may occur [55]. Therefore, a proper selection of the matrix material while preparing the phosphor-based color conversion layers is a way to significantly improve the efficiency of the generated white light.

## Conclusion

A facile, water-based, rapid synthesis method for the fabrication of lanthanide-doped hydroxide-based phosphors at room temperature is presented. Introducing acetate-based metals and lanthanides into the LiOH/water solvent system (specifically, yttrium and europium acetates, for this study) demonstrated rapid crystallization of lanthanide-doped hydroxides. These crystals were driven by a crystal splitting growth mechanism that leads to the formation of a multipod structure. Furthermore, this method allows the controlled doping ratio by varying the dopant concentration. The fabricated red-emitting Y(OH)_3_:Eu^3+^ phosphors are integrated into well-known YAG-based color conversion layers in order to generate white light. For future applications, this study may lead to fabrication of various phosphors doped with different lanthanides that can result in a targeted color emission. In this sense, one can produce a white LED system composed of a UV-LED chip with red–green–blue phosphors since the method presented here offers an easy, inexpensive and environmentally friendly synthesis for the fabrication of these phosphors.
